# Early Syllabic Segmentation of Fluent Speech by Infants Acquiring French

**DOI:** 10.1371/journal.pone.0079646

**Published:** 2013-11-07

**Authors:** Louise Goyet, Léo-Lyuki Nishibayashi, Thierry Nazzi

**Affiliations:** 1 Université Paris Descartes, Paris, France; 2 CNRS Laboratoire de Psychologie de la Perception, Paris, France; Lancaster University, United Kingdom

## Abstract

Word form segmentation abilities emerge during the first year of life, and it has been proposed that infants initially rely on two types of cues to extract words from fluent speech: Transitional Probabilities (TPs) and rhythmic units. The main goal of the present study was to use the behavioral method of the Headturn Preference Procedure (HPP) to investigate again rhythmic segmentation of syllabic units by French-learning infants at the onset of segmentation abilities (around 8 months) given repeated failure to find syllabic segmentation at such a young age. The second goal was to explore the interaction between the use of TPs and syllabic units for segmentation by French-learning infants. The rationale was that decreasing TP cues around target syllables embedded in bisyllabic words would block bisyllabic word segmentation and facilitate the observation of syllabic segmentation. In Experiments 1 and 2, infants were tested in a condition of moderate TP decrease; no evidence of either syllabic or bisyllabic word segmentation was found. In Experiment 3, infants were tested in a condition of more marked TP decrease, and a novelty syllabic segmentation effect was observed. Therefore, the present study first establishes early syllabic segmentation in French-learning infants, bringing support from a syllable-based language to the proposal that rhythmic units are used at the onset of segmentation abilities. Second, it confirms that French-learning infants are sensitive to TP cues. Third, it demonstrates that they are sensitive to the relative weight of TP and rhythmic cues, explaining why effects of syllabic segmentation are not observed in context of high TPs. These findings are discussed in relation to theories of word segmentation bootstrapping, and the larger debate about statistically- versus prosodically-based accounts of early language acquisition.

## Introduction

Infants acquiring language have to learn about the phonology, the lexicon and the syntax of their native language. The present study explores some of the mechanisms involved in learning the lexicon, namely the ability to extract the sound pattern of words from fluent speech (henceforward, word form segmentation). Word form segmentation constitutes a crucial step in lexical acquisition, because most speech directed to infants is constituted of multiword utterances [1-3] and its importance for learning words is supported by findings of links between early word segmentation and later vocabulary learning [4,5]. Many studies have explored the emergence of word form segmentation, using either a behavioral method (HeadTurn Preference Procedure, or HPP) or an electrophysiological method (Evoked-Response Potentials, or ERPs), and have established that this ability emerges between 6 and 12 months of age in infants learning English [6,7], Parisian French [8-10], Canadian French [11,12], Dutch [13] and German [14]. The goal of the present study is to determine the procedures that infants use to segment speech when this ability emerges, focusing on French-learning infants in order to establish early rhythmic-based segmentation and to directly explore the relative importance of rhythmic and distributional information.

Previous research has established that during the first year of life, infants use many subtle linguistic cues present in the signal to segment fluent speech. These cues include transitional probabilities (TPs) between syllables [15-18], the rhythmic unit of the native language [8,9,19,20], prosodic boundaries [21,22], co-articulatory cues [15], allophonic information [23,24], phonotactic information [25-27] and pitch accent [28].

The first two cues (TPs and rhythmic units), which are further explored in the present study, have received the most attention and have been proposed as crucial at the onset of word segmentation [7,9,23]. This interest initially stems from the fact that they are both seen as instantiations, at the level of early word form segmentation, of the debate between two dominant visions of language acquisition. On the one hand, the use of TPs is linked to the proposal that infants are able to perform sophisticated statistical analyses of the speech signal that will allow them to discover many properties of their native language, such as its phoneme inventory [29], its lexicon [17], or aspects of its syntactic properties [30,31]. On the other hand, the use of rhythmic unit cues is linked to “prosodic or phonological bootstrapping” theories [32] claiming that the speech signal contains many acoustic/prosodic cues that also allow infants to learn properties of their native language, such as its basic rhythm [33], its lexicon and some properties of that lexicon [20,34,35], or its syntactic structure [36,37]. While these two theoretical perspectives were initially considered to be in opposition [15-18], a more recent position is that they might both contribute to language acquisition, and current work is trying to understand how the two types of cues are used in combination (e.g., [16,38,39]). The present project contributes to this debate.

Hence, in the context of early segmentation, it has been proposed that the combined use of (statistical) TPs that are taken to be language-general and rhythmic units that differ across classes of languages could account for early differences in segmentation abilities across languages, in particular between English-learning infants relying on trochaic units and French-learning infants relying on syllabic units. However, the evidence for the early use of syllabic units is limited as research on Parisian French has repeatedly failed to find evidence in its favor before 12 months [9,10] while syllabic effects found in Canadian French 8-month-olds [11] might come from post-lexical processes rather than segmentation processes. This limited evidence thus weakens the rhythmic unit proposal in general (see more below). The present study re-explores this issue, and examines how combined use of rhythmic units and TP information might explain previous failures to show early syllabic segmentation in Parisian infants. Before presenting the study itself, we review in more detail the literature on segmentation based on TPs and rhythmic units.

Regarding distributional information, most studies used the HPP to explore infants’ ability to use transitional probabilities between adjacent syllables (TPs), which refer to regularities in the order of syllables in the speech signal (for a pair of events ‘xy’, forward TPs measure the strength with which ‘x’ predicts ‘y’ while backward TPs measure the strength with which ‘y’ predicts ‘x’), and appeared important given that TPs are higher within than across words [19]. By using an artificial language paradigm in which infants are presented with a continuous sequence made-up of randomly ordered repetitions of 4 trisyllabic pseudo-words, English-learning 6- and 8-month-olds were found to group syllables into cohesive word-like units on the basis of syllabic distributional information [7,18,40], a finding later extended to natural language situations [41,42]. Similar results were found at the same age in Dutch-learning [43] and French-learning [16] infants. Interestingly, the use of distributional cues appears to be wide ranging, and was found in non-human primates [44] and for the perception of musical sequences by human infants [17]. However, some limits in infants’ ability to use TPs for segmentation have been found when using more complex languages [15,16,43]. Therefore, current research is exploring how TPs are used in conjunction with other cues in more complex experimental situations [16,18,41,42] an issue that will also be addressed here.

The use of the rhythmic (prosodic) unit of the native language has been formalized in the early rhythmic segmentation hypothesis [9]. This hypothesis states that infants use the rhythmic unit on which the rhythm of their native language is based to segment the continuous speech stream. Given that languages have different rhythmic units (the syllable in syllable-based languages such as French and Italian; the trochaic or strong-weak stress unit in stress-based languages such as English, Dutch and German [45,46]), this hypothesis predicts that there should be cross-linguistic differences in the way segmentation mechanisms will be established during development.

Evidence for the rhythmic-based hypothesis initially comes from studies on stress-based languages. These HPP studies suggest that after having developed a bias for trochaic stress units ([34] for English [36]; for German), English-learning infants use this rhythmic information by 8 months of age to segment fluent speech into trochaic units [15,19,20,28,47-49]. For example, after having been familiarized with repetitions of trochaic target bisyllabic words (uttered in isolation), 7.5-month-old infants show a preference for passages containing the target bisyllabic words presented in a test phase. However, they do not show a preference for the same passages after having been familiarized with only the stressed syllables of the trochaic words, suggesting they do not extract and then recognize the individual stressed initial syllables from the trochaic words [20]. These findings using the word-passage order (familiarization with isolated targets and test with passages containing or not containing the targets) were replicated in the passage-word order (in which the order of presentation of the stimuli between familiarization and test is reversed, hence a familiarization with passages and test with isolated targets and controls) [20]. Convergent findings have also been obtained in young Dutch-learning infants using HPP [13,50] and ERPs [50-52]. Therefore, results on English- and Dutch-learning infants are compatible with the notion that the trochaic unit is used at the onset of word form segmentation by infants learning stress-based languages.

For syllabic-based segmentation in syllable-based languages, all existing research focuses on French-learning infants. However, the studies have not provided any clear evidence of syllabic segmentation at the onset of this ability around 8 months of age. Most studies on this topic have tested Parisian infants learning European French. While [53] found that 7.5-month-old Parisian infants can segment monosyllabic words in the word-passage order, it should be noted that this does not establish the role of the syllabic unit given that the lexical and syllabic levels are confounded in monosyllabic words. In order to establish syllabic segmentation, one needs to show that infants can segment syllables embedded in multisyllabic words. Accordingly [9], investigated if infants can segment bisyllabic words or each of their individual syllables from passages, after having been familiarized with lists of either the bisyllabic target words or one of their syllables (word-passage order). Their results failed to find any evidence of segmentation at 8 months, but established syllabic segmentation at 12 months (with stronger evidence for the segmentation of the word-final syllables), and bisyllabic segmentation at 16 months. This pattern of results suggests a precedence in developmental time of syllabic over whole (multisyllabic) word segmentation in French, in accordance with the syllable-based segmentation hypothesis.

However, this precedence of syllabic segmentation was challenged by follow-up studies using different methods (HPP or ERPs) and variants of the procedure (use of the word-passage or passage-word orders). Indeed, results using ERPs suggested that Parisian 12-month-olds could access both the syllabic and the whole word levels [8]. Moreover, when tested in the word-passage order, Canadian French 8-month-olds were found to recognize not only the target bisyllabic words (familiarity preference), but also their initial syllables (novelty preference) and marginally their final syllables (novelty preference) [11]. Even more problematic for the syllable-based segmentation hypothesis, a study using the stimuli by [11] and the passage-word order (familiarization with passages, test with either the target bisyllabic words or their final syllables) found evidence of bisyllabic word segmentation (while replicating the lack of evidence of bisyllabic word segmentation in the word-passage order), but no such evidence for their final syllables in Parisian 8-month-olds [10]. These last findings suggest that both Parisian and Canadian French-learning infants can segment bisyllabic words at 8 months. While there is no evidence that Parisian 8-month-olds segment syllables, the results by [11] suggest that Canadian 8-month-olds might also segment individual syllables. However, the fact that [11] found a novelty rather than a familiarity effect when looking at syllabic segmentation calls for a replication. Moreover, it is unclear from their study whether the recognition of the target syllable preceded the recognition of the bisyllabic word it was embedded in, or whether it was the segmentation and recognition of the bisyllabic word that allowed the later recognition of the syllable. In the latter case, the observed syllabic effect would not be an effect of segmentation, but rather a product of “post-lexical” processing.

The above findings thus seem contrary to the prediction of syllable-based segmentation, and therefore appear to invalidate the early rhythmic segmentation hypothesis. If so, a more parsimonious interpretation of the findings so far might be that TPs are the primary cues to early word segmentation in all languages and that prosody is only a secondary cue that would play a role only in language-specific ways – in the present case, only in languages in which there is a clear trochaic bias in the lexicon. However [10], proposed a way in which their findings could be reconciled with syllable-based segmentation, and hence, with the early rhythmic segmentation hypothesis, which is tested in the present study. The idea is that 8-month-old Parisian infants use both the rhythmic unit of their native language (the syllable) and TPs to segment words. Evidence of bisyllabic word segmentation in their study was only found in the passage-word order because in that order, infants have time during familiarization to segment syllabic units and conduct some analyses to retrieve distributional information (whether forward or backward TPs, all equal to 1 in the stimuli used) that will allow them to group together the two syllables of the bisyllabic words. As a corollary, the use of the syllabic unit was masked because the syllables infants had to detect were always part of bisyllabic words, so that high TPs induced grouping of the two syllables of the target words.

Therefore, the goal of the present study was to reevaluate syllabic segmentation in light of this hypothesis by testing Parisian 8-month-old infants in the passage-word order and, crucially, using passages in which the strength of the distributional (TP) cues was gradually manipulated. In all previous studies exploring syllabic segmentation [9-11], forward and backward TPs between the two syllables of the bisyllabic words were equal to 1, because the two syllables of the target words always appeared together. In the present study, TP information was reduced by selecting, for each target syllable, several words sharing that syllable. Two levels of decreased distributional information were used, a moderate one in which each target syllable was shared by two bisyllabic words (e.g., *diva/radis*; Experiments 1 and 2), and an even more marked decrease in which each target syllable was shared by 8 different words (Experiment 3). While [10] found a pattern of results in which bisyllabic words, but not their individual syllables, were found to be segmented, we predicted that the present manipulations should reverse this pattern, and allow us to observe segmentation of syllables, but not bisyllabic words. Syllabic segmentation was tested for both manipulation levels (Experiments 1: moderate decrease and 3: marked decrease), and bisyllabic word segmentation only for the moderate level (Experiment 2).

## Experiment 1

 The aim of Experiment 1 was to reevaluate syllabic segmentation in (Parisian) French-learning 8-month-olds in a context of moderate reduction of distributional information around the target syllables. Accordingly, infants were familiarized with two passages (each made up of 8 sentences), each corresponding to a different target syllable (e.g., /di/ and /pu/), and each containing two bisyllabic words sharing that target syllable in either word-initial or word-final position (e.g., /diva/-/radi/ for the /di/ syllable). Then, they were tested with two lists of the repeated target syllables and two lists of repeated control syllables. In this context, the forward TPs for the words with the target syllables in word-initial versus word-final positions were .5 and 1 respectively, while the backward TPs for the same words were 1 and .5. We predicted that if such a moderate reduction of TP information within the bisyllabic words compared to [10] is enough to give less weight to TP- relative to rhythmic-based segmentation, then French-learning 8-month-old infants might show a syllable-based segmentation effect in the present experiment, and no bisyllabic word segmentation effect in Experiment 2.

### Method

#### Ethics statement

Parents of all infant participants provided written informed consent prior to the experiment. The experimental protocol and consent procedure were both approved by the CERES (Comité d’évaluation éthique des projets de recherche) of the Université Paris Descartes. All data were obtained according to the principles expressed in the Declaration of Helsinki.

#### Participants

Twenty 8-month-old infants (mean age: 8.3 months; range: 7.8-8.8 months) from French-speaking families were tested (9 females and 11 males). Data from four additional infants were excluded from the analysis due to fussiness (3) or due to a segmentation index (defined as the difference between the mean orientation times to the lists of target and control syllables) more than 2 SDs above or below the group mean (1). All infants were full term, had no major problem during pregnancy and birth and had normal development and hearing. Their parents had neither hearing impairment nor language problems. The infants were recruited through birth lists from the Paris area and parents gave a written informed consent.

#### Stimuli

As done in previous research on this topic, we selected stimuli with relatively low frequencies, using the adult database LEXIQUE [54] (given per 1 million occurrences, and calculated over a base of 31 million occurrences). We selected four syllables with relatively low frequencies in the initial and final positions of French words: /ba/ (initial position: 5.99; final position: 14.12); /di/ (initial position: 16.10; final position: 5.94); /pu/ (initial position: 5.19; final position: .09) and / /tɔ~/ (initial position: 62.84; final position: 7.48). For each syllable, two target words that start or end with that syllable were also chosen for their relatively low frequencies: syllable /ba/: *bassin-tuba* (/basῖ/-/tyba/, [pool-tuba]; word frequency: .07 and .54, respectively); syllable /di/: *diva-radis* (/diva/-/radi/, [diva]-[raddish]; word frequency: 1.28 and 3.11, respectively) syllable /pu/: *poulain-quipou* (/pulῖ/-/kipu/, [young horse]-[quipou]; word frequency: 3.04 and .07, respectively); syllable /tɔ~/: *tombeau-jeton* (/tɔ̃bo/-/ʒətɔ~/, [grave]-[chip]; word frequency: 11.96 and 10.27, respectively). There was no semantic link between the target words sharing the same syllable. All these items are likely to be unknown to the infants tested given their relatively low frequencies in the adult lexicon LEXIQUE, and the fact that they do not appear in the infant (8-16 months) version of the French MCDI [55], a checklist of French words acquired at an early age, except for “ton”, which however is known by 0% of infants at 8 months. 

Lastly, for each syllable, four passages (sentences) were created, each made up of eight sentences. In each passage, half of the sentences contained a bisyllabic target word, and the other half contained the other bisyllabic target word sharing the same target syllable. The target syllables in word-initial positions were always preceded by different syllables; similarly, the target syllables in word-final positions were always followed by different syllables (see Appendix S1). There was no semantic link between the sentences in each passage (as done in previous studies, e.g. [6] for English [9], for French). 

Recordings were made in a sound-attenuated booth. A female native speaker of French first recorded the four passages. She was asked to produce the stimuli as if speaking to an infant. All the passages were 20 s long and the sentences were separated by a mean ISI of 560 ms. The target syllables in the passages had an average duration of 155 ms in initial position and 154 ms in final position. Their mean intensity was 72 and 70 dB respectively, and their mean pitch was 230 and 236 Hz respectively.

For each target syllable, the same talker also produced a list of 20 isolated occurrences for use in the test phase. The talker produced the tokens with some variation, as in previous research (e.g., [6] for English [9], for French), in order to prevent infants’ boredom when listening to the test lists, and also to evaluate recognition of the targets at test in a condition with some acoustic variability. The duration of each list was 20 s and the syllables were separated by a mean ISI of 740 ms. The target syllables in the lists had an average duration of 275 ms, a mean intensity of 76 dB and a mean pitch of 224 Hz.

#### Procedure, apparatus and design

The experiment was conducted in a sound-attenuated booth, which contained a three-sided test booth made of pegboard panels. The test booth had a red light and loudspeakers (Sony xs-F1722) mounted on each of the side panels and a green light mounted on the central panel. Directly below the center light, a 5-cm hole accommodated the lens of a video camera used to monitor infants’ behavior. A PC computer terminal (Dell Optilex computer), audio amplifier (Marantz PM4000), TV screen and response box were located outside the sound-attenuated room. The stimuli were stored in digitized form on the computer and were delivered by the loudspeakers via the amplifier. The response box, which was connected to the computer, was equipped with 3 buttons. The box was controlled by an observer, outside the sound-attenuated booth, who watched the video of the infant on the TV screen and pressed the buttons according to the direction the infant’s headturns, thus starting and stopping the flashing of the lights and the presentation of the sounds. The observer and the infant’s caregiver wore earplugs and listened to masking music over tight-fitting headphones, which prevented them from hearing the stimuli. Information about the direction and duration of the headturns/orientation times were stored in a data file on the computer.

The variant of HPP set up by [6] was used in the present experiment. Each infant was held on a caregiver’s lap and the caregiver was seated in a chair at the center of the test booth. Each trial began with the green light on the center panel blinking until the infant had oriented in that direction. Then the center light was extinguished and the red light above the loudspeaker on one of the side panels began to flash. When the infant made a turn of at least 30° in the direction of the loudspeaker, the stimulus for that trial was played, the red light continuing to flash for the entire duration of the trial. Each stimulus was played to completion (i.e., when the eight sentences of a given passage had been presented) or stopped immediately after the infant failed to maintain the 30° headturn for two seconds. If the infant turned away from the red light for less than two seconds and then turned back again, the trial continued but the time spent looking away was not included in the orientation time. Thus, the maximum orientation time for a given trial was the duration of the entire speech sample. If for a trial, the infant’s orientation time was shorter than 1.5 seconds, the trial was immediately replayed and the initial orientation time was discarded.

Each experimental session began with a familiarization phase in which infants heard two passages on alternating trials until they accumulated 30 s of orientation times to each. When the infants reached the familiarization criterion for one passage, the second passage continued to be presented until its criterion was also reached. The side of the loudspeaker from which the stimuli were presented was randomly varied from trial to trial. The test phase began immediately after the familiarization criterion was reached. It consisted of three test blocks, in each of which the four lists of isolated syllables were presented. The order of the lists within each block was randomized.

Half of the infants were familiarized with the /ba/ and /tɔ̃/ passages (Condition 1), and the other half with the /di/ and /pu/ passages (Condition 2). All the infants were tested with the four lists of syllables. Therefore, the target syllables were /ba/ and /tɔ̃/ for the infants in Condition 1, and /di/ and /pu/ for the infants in Condition 2.

### Results and Discussion

**Figure 1:**
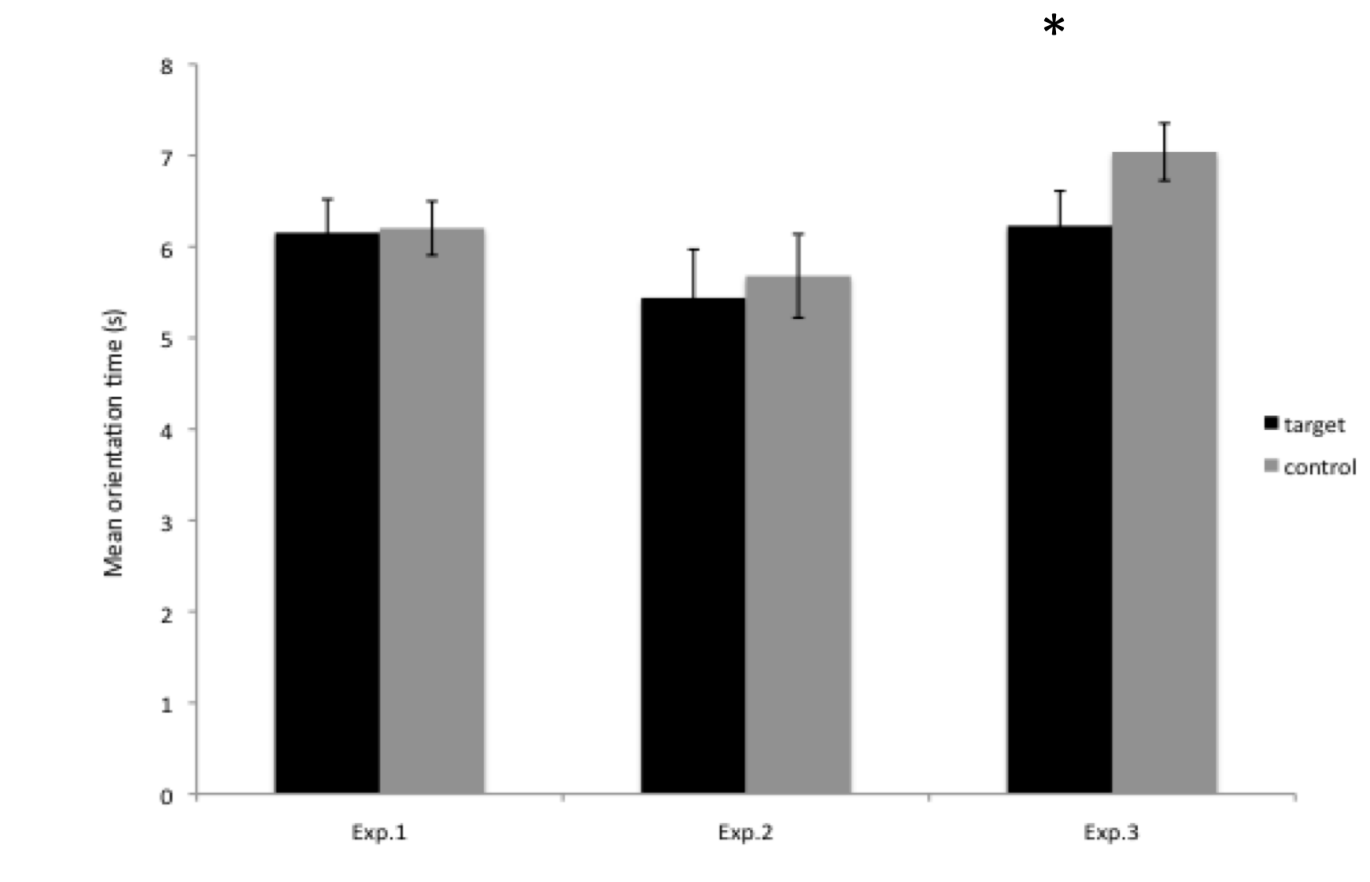
Mean orientation times (in seconds) to the target vs. **control syllables in Experiments 1 - 3.** The error bars indicate the standard error of the mean.

The analyses were conducted following the same data analyses as done in previous studies using this kind of segmentation paradigm (e.g. [6] for English [9,10], for French). Mean orientation times to the lists containing the target versus the control syllables were calculated for each infant (see Figure 1, left panel). In this kind of study, differences in orientation times to the target versus the control test stimuli are taken as evidence that infants have segmented the targets from the passages and recognized them in the test phase. Therefore, a 2-way ANOVA with the main between-subject factor of Condition (Conditions 1-2) and the main within-subject factor of Familiarity (lists of target syllables heard during familiarization versus lists of control syllables not heard during familiarization) was conducted. The effect of Familiarity did not reach significance (F(1,18) = .02, p = .86, η^2^ = .001), indicating that infants had similar orientation times to the target (M = 6.15 s, SD = 1.64) and control (M = 6.20 s, SD = 1.33) syllables. Moreover, only 9 of the 20 infants oriented longer to the familiar lists (binomial test, p = .74). Neither the effect of Condition (F(1,18) = 1.76, p = .19 ) nor the Familiarity x Condition interaction (F(1,18) = 1.77, p = .19) reached significance.

Experiment 1 fails to reveal a syllabic segmentation effect at 8 months of age and is thus in line with previous studies that failed to find syllabic effects in young Parisian French-learning infants [9,10]. This failure is found in spite of the fact that we have moderately decreased the relative weight of TP information by associating each target syllable with two bisyllabic words (e.g., for the syllable /di/: /diva/-/radi/). One possible reason for the failure to segment syllables is that TPs were still too high (moderate decrease) between the syllables of the target bisyllabic words, and that infants, as in [10] were segmenting the bisyllabic target words rather than their individual syllables. This possibility was explored in Experiment 2, in which infants were familiarized with the same passages as in Experiment 1, but then tested on their recognition of the whole bisyllabic words and not on their isolated syllables.

## Experiment 2

The aim of Experiment 2 was to evaluate whether French-learning 8-month-olds segment bisyllabic words rather than individual syllables from the passages used in Experiment 1, thus in a context of moderate decrease of TP information. Accordingly, they were familiarized with two passages, each corresponding to a different target syllable (e.g., /di/ and /pu/), and each containing two bisyllabic words sharing that target syllable in either word-initial or word-final position (e.g., /diva/-/radi/ for the /di/ syllable), and then tested with two lists of the target bisyllabic words and two lists of control words.

### Method

#### Participants

 Twenty 8-month-old infants (mean age: 8.2 months; range: 7.7-8.7 months) from French-speaking families were tested (13 females and 7 males). Data from four additional infants were excluded from the analysis due to fussiness (4). Infants were recruited following the same procedure and criteria as in Experiment 1. 

#### Stimuli

The familiarization stimuli were the same passages as in Experiment 1. The pair of target words in the passages had an average duration of 340 ms. Their mean intensity was 71 dB, and their mean pitch was 232 Hz. The female talker of Experiment 1 had also recorded four lists of 20 alternating target words, again as if speaking to an infant, and with some variation, one for each target syllable, which were used in the present test phase. The duration of each list was 20 s and the words were separated by a mean ISI of 560 ms. The target words in the lists had an average duration of 482 ms, a mean intensity of 70 dB and a mean pitch of 200 Hz.

#### Procedure, apparatus and design

The procedure and apparatus were identical to Experiment 1. Half of the infants were familiarized with the /ba/ and /tɔ̃/ passages (Condition 1), and the other half with the /di/ and /pu/ passages (Condition 2). All the infants were tested with the four lists of pair of bisyllabic words.

### Results and Discussion

As for the test phase of Experiment 1, mean orientation times to the lists containing the target versus the control words were calculated for each infant (see Figure 1, middle panel). A 2-way ANOVA with the between-subject factor of Condition (Conditions 1-2) and the within-subject factor of Familiarity (lists of target words heard during familiarization versus lists of control words not heard during familiarization) was conducted. The effect of Familiarity did not reach significance (F(1,18) = .022, p = .64, η^2^ = .011), indicating that infants had similar orientation times to the target (M = 5.44 s, SD = 2.13) and control (M = 5.68 s, SD = 1.84) words. Moreover, only 10 of the 20 infants oriented longer to the familiar lists (binomial test, p = .58). Neither the effect of Condition (F(1,18) = 0.75, p = .39) nor the Familiarity x Condition interaction (F(1,18) = .62, p = .43) reached significance.

Experiment 2 failed to provide evidence that infants were segmenting whole bisyllabic words from the passages. These results differ from [10], who showed using the same procedure that French-learning 8-month-olds are able to segment bisyllabic words. One possible reason for the present lack of a segmentation effect could be the alternation between the two target words in both the familiarization and the test phases, which could have made the task more difficult for the infants. More interestingly, the lack of bisyllabic word segmentation in Experiment 2, paired with the lack of syllabic segmentation in Experiment 1, is likely to be due to our TP manipulation. It is possible that having moderately decreased the TPs around the target syllables by embedding them in two rather than one word was enough to block the segmentation of the bisyllables without allowing us to observe syllabic segmentation. Since finding evidence of syllabic segmentation at 8 months in Parisian French-learning infants is the main focus of the present study, Experiment 3 was conducted to evaluate this issue in a context of even more decreased TP cues.

## Experiment 3

 The aim of Experiment 3 was again to assess syllabic segmentation in French-learning 8-month-olds, in a context of even lower TPs (marked decrease) than in the passages used in Experiments 1 and 2. Accordingly, French-learning 8-month-olds were familiarized with new passages, each containing 8 different target words that all shared a target syllable. For example, for the passage corresponding to the target syllable /di/, half of the words contained the target syllable in word-initial position (*divan, dizain, dîner, dito*) while the other half contained the same target syllable in word-final position (*bandit, taudis, caddie, radis*; all target syllables being pronounced as /di/ no matter the spelling, see stimuli section below). Thus in Experiment 3, the forward TPs for the words with the target syllables in word-initial versus word-final positions were .125 and 1 respectively, while the backward TPs for the same words were 1 and .125. We predicted that if this much more drastic reduction of TP information within the bisyllabic words compared to Experiment 1 is enough to cause infants to give less weight to TP- relative to rhythmic-based segmentation, then French-learning 8-month-old infants might show a syllable-based segmentation effect in this third experiment.

### Method

#### Participants

Twenty 8-month-old infants (mean age: 8.3 months; range: 7.9-9.2 months) from French-speaking families were tested (7 females and 13 males). Data from one additional infant were excluded from the analysis due to fussiness. Infants were recruited following the same procedure and criteria as in Experiments 1 and 2.

#### Stimuli

In this experiment, three of the four syllables chosen as stimuli differed from the ones used in Experiment 1 because eight relatively low frequency words per syllable needed to be selected, and this was only possible for one of the syllables used in Experiment 1. Therefore, we again used Lexique [54] to select four syllables with relatively low frequencies (again given per 1 million of occurrences) in initial and final positions of French words: /di/ (initial position: 11.39; final position: 4.56), /gu/ (initial position: 2.01; final position: 6.52), /po/ (initial position: 6.87; final position: 6.15) and /te/ (initial position: 3.7; final position: 11.95). For each syllable, eight words were chosen for their low frequencies. For example, for the syllable /di/, we used: *divan* (/divã/, [couch], 21.55), *dizain* (/dizῖ/, [dizain], .00), *diner* (/dine/, [dinner], 60.0), *ditto* (/dito/, [ditto], .34), *bandit* (/bãdi*/*, [outlaw], 4.59), *taudis* (/todi*/*, [hovel], 3.24), *caddie* (/cadi/, [caddy], 1.28), *radis* (/radi/, [radish], 3.11). There was no semantic link between the 8 words sharing a given syllable. All these items are likely to be unknown to the infants tested given their relatively low frequencies in the adult lexicon LEXIQUE, and the fact that they do not appear in the infant (8-16 months) version of the French MCDI [55], except for “pot”, which however is known by only 3% of infants at 8 months.

Lastly, four passages were created for each target syllable (passages /di/, /gu/, /po/, /te/). Each passage was made up of 8 sentences (one for each of the 8 bisyllabic words) in which the target syllable was in initial position of four words and in final position of the other four words. The target syllables in word-initial positions were always preceded by different syllables; similarly, the target syllables in word-final positions were always followed by different syllables (see Appendix S2). There was no semantic link between the different sentences in each passage (as done in previous studies, e.g. [6] for English [9], for French).

As in Experiments 1 and 2, recordings were made in a sound-attenuated booth. The same female talker recorded the four passages. The talker was again asked to produce the stimuli as if speaking to an infant. All the passages were 20 s long and the sentences were separated by an ISI of 480 ms. The target syllables in the passages had an average duration of 133 ms in initial position and 147 ms in final position. Their mean intensity was 74 and 70 dB respectively, and their mean pitch was 216 and 227 Hz respectively.

For each target syllable, the same talker also produced a list of 20 isolated occurrences for use in the test phase. Again, the talker produced the tokens with some variation. The duration of each list was 20 s and separated by a mean ISI of 590 ms. The target syllables in the lists had an average duration of 255 ms, a mean intensity of 74 dB, and a mean pitch of 217 Hz.

#### Procedure, apparatus and design

The procedure and apparatus were identical to Experiment 1. Half of the infants were familiarized with the passages /di/ and /po/ (Condition 1) and the other half with the passages /gu/ and /te/ (Condition 2). All the infants were tested with the same four lists of syllables. Therefore, the target syllables were /di/ and /po/ for the infants in Condition 1, and /gu/ and /te/ for the infants in Condition 2.

### Results and Discussion

Again, mean orientation times to the lists containing the target versus the control syllables were calculated for each infant (see Figure 1, right panel). A 2-way ANOVA with the between-subject factor of Condition (Conditions 1-2) and the within-subject factor of Familiarity (lists of target syllables heard during the familiarization phase versus lists of control syllables not heard during the familiarization) was conducted. The effect of Familiarity was significant (F(1,18) = 5.09, p = .03, 64, η^2^ = .220, large effect), indicating that infants had a preference for the control (M = 7.04 s, SD = 1.4) over the target (M = 6.22 s, SD = 1.71) syllables. Moreover, 15 out of 20 infants oriented longer to the control than to the target lists (binomial test, p = .02). Neither the effect of Condition (F(1,18) = 1.12, p = .30), nor the Familiarity x Condition interaction (F(1,18) = .07, p = .78) reached significance.

The present results show that Parisian French-learning 8-month-olds, familiarized with passages containing target syllables, react differently to presentation of repetitions of these syllables versus control syllables during the test phase. The present findings are thus the first to directly establish sensitivity to the syllabic unit at the brink of word form segmentation, as predicted for syllable-based languages such as French by the early rhythmic-based segmentation hypothesis. This result thus demonstrates syllabic segmentation effects earlier than had previously been found ([8,9] for 12-month-olds).

Before further discussing in the General Discussion why such a clear syllabic segmentation effect had not been observed in past studies at 8 months of age, we would like to address one feature of our finding, namely that the segmentation effect corresponded to a novelty effect (infants preferred to orient to the control syllables over the familiar syllables) rarely found in segmentation studies. Interestingly, this novelty effect was also found in [11] when they tested whether Canadian French infants familiarized with the initial or final syllables of bisyllabic words would later listen differently to passages containing these bisyllables and to control passages (while they found a familiarity effect for bisyllabic word segmentation). However, familiarity effects for monosyllabic word segmentation in Parisian 8-month-olds were found in both the word-passage [53] and passage-word orders (unpublished data from our group). Taken together, these findings raise the possibility that these novelty effects are due to the fact that infants are reacting differently according to whether the target syllables correspond to a word or are part of a word. This suggests that French-learning infants might be sensitive to word boundary cues or coarticulation factors that are stronger within words than across word boundaries, as found for young English-learning infants [15,56].

## General Discussion

The main goal of the present study was to provide evidence for early syllabic segmentation in French-learning infants, as predicted by the early rhythmic segmentation hypothesis for syllable-based languages like French. More precisely, we re-evaluated French-learning 8-month-olds’ ability to segment syllabic units, given that such effects have never been found before 12 months in Parisian infants ([8-10]; see below for a discussion of the results by [11] with Canadian French infants), in two experimental contexts varying in the distributional properties of the passages to be segmented. While the results of Experiments 1 and 2 revealed neither syllabic nor whole word segmentation (in a context of moderate TP decrease), a syllabic segmentation was found in Experiment 3 in which the decrease in TPs was more pronounced.

Interestingly, in spite of methodological differences (different stimuli, different test orders, etc.), our results converge with those of [11] that found in the word-passage order that Canadian French-learning 8-month-olds familiarized with passages containing bisyllabic words recognize both the bisyllables (familiarity effect) and their individual syllables (novelty effects, marginal for final syllables, significant for initial syllables). These previous findings suggested that Canadian French-learning 8-month-olds were also accessing the syllabic level, together with the lexical level. However, as we argued earlier, it was unclear from their study whether the recognition of the target syllables preceded the recognition of the bisyllabic words they were embedded in, or whether it was the segmentation and recognition of the bisyllabic words that allowed the later recognition of the syllables (thus, a post-lexical effect in this latter case). This ambiguity does not arise with our study. Indeed, if the present syllabic effect were the reflection of post-lexical processing, this would mean that infants in Experiment 3 would have had access to each target syllable after having segmented (some of) the eight bisyllabic words per passage in which it was embedded. This is likely a very difficult task at this age. Moreover, if it were the case, it would be intriguing that they would not have done the same in Experiment 1, given that there were only two target words per passage; however, Experiment 2 provides no evidence that 8-month-olds were indeed segmenting the bisyllabic words in the passages used in both Experiments 1 and 2. Therefore, a more parsimonious interpretation of the finding of Experiment 3 is that it shows that Parisian French-learning infants can segment syllabic units from fluent speech at 8 months, but that this can only be observed under specific experimental conditions.

Our study, together with [10], allows us to specify the conditions under which syllabic segmentation is observed (passage-word order: present Experiment 3) or not (word-passage order [9]:; passage-word order [10]:, present Experiment 1) at 8 months. First, the main difference between Experiment 3 and [9] is a change in stimulus order, so that in the test phase, when their behavior was measured, infants were hearing lists of isolated syllables rather than passages. Together with the finding that infants can segment bisyllabic words in the passage-word but not the word-passage order [9,10], it appears that it is easier for Parisian French-learning infants to show evidence of segmentation in the passage-word order. It was suggested that this might in part be due to them having more time to process the passages in the passage-word than word-passage order [10].

Second, and more importantly, the results of the present Experiment 3 differ from those of [10] and our Experiment 1, both of which tested infants in the same passage-word order. The crucial difference between these three experiments relates to the strength of TPs within the passages, which was maximal in [10], moderate in Experiment 1, and lowest in Experiment 3. Taken together, these experiments thus establish not only that French-learning 8-month-olds use syllables to segment speech, but also that they use TP information (confirming data from [16], obtained in an artificial language paradigm), and that infants are sensitive to the relative strength of the two cues. This is attested by the fact that a syllabic effect is only observed when TP cues are the weakest (Exp. 3), but not when they are moderate (Exp. 1) or high (as in [10]). When syllabic segmentation is not observed, we suggest that it does occur in a first segmentation step, but because infants also process TP information regarding the fact that certain syllables are likely to be part of a bisyllabic unit, the recognition of the syllable is blurred by the formation of the bisyllabic unit. Note that the formation of the bisyllabic unit is also influenced by the strength of the TP cue: while high TPs led to formation and recognition of the bisyllabic targets in [10], the moderate decrease in TP cues of the stimuli used for the present Experiments 1 and 2 probably did not allow the formation of stable enough bisyllabic units, leading to a lack of recognition of these units in Experiment 2.

Therefore, the present findings are the first ones to demonstrate that French-learning infants use the rhythmic unit of their native language (the syllable) to segment fluent speech at the time in development when this ability emerges (while confirming it is not observed under every condition). They complement previous findings on two stress-based languages, English and Dutch, which had established that these infants use the rhythmic unit of their native language, the trochaic stress unit, when segmentation emerges [20,51,52]. Our results thus bring crucial support, from a language of a different rhythmic class, to the early rhythmic-based segmentation hypothesis that has been proposed to be essential for the emergence of segmentation procedures [9]. This piece of evidence is crucial since it validates this general proposal, rather than the more restricted interpretation of the English and Dutch data presented earlier (according to which the rhythmic cue is a secondary cue, used only in some languages with trochaically-biased lexicons, while TPs would be the primary, default, language-general segmentation cue). Our findings thus establish the early use in segmentation of two different rhythmic units (the syllabic unit, the trochaic units), used by infants appropriately according to the rhythmic properties of their native language. What future work will have to specify is how the specific rhythmic unit of the native language is acquired before the onset of segmentation abilities. One proposal is that this acquisition might rely on newborns’ sensitivity to the global rhythm of languages and on infants’ rapid acquisition of the native rhythm of their native language by 5 months of age [33,57]. Our data, by supporting both a prosodically-based and a statistically-based account of early segmentation, contribute to the current shift in the field of language acquisition according to which both statistically-based and prosodic/phonological bootstrapping theories account for part of early language acquisition, from which it follows that the central issue nowadays is to understand how the use of the two kinds of cues interacts in language acquisition.

With respect to the statistical cue of transitional probabilities (TPs), our results also confirm the early use of TPs by young French-learning infants. Indeed, the comparison of the outcomes of Experiments 1 and 3 shows that the syllabic effect is found when within-word TPs are low (as low as .125, Experiment 3) but not when they are 4 times (.5, Experiment 1) or 8 times higher [10]. This finding first confirms prior results using the artificial language paradigm that Parisian French-learning infants are sensitive to TPs by 8 months of age [16], just like English- and Dutch-learning infants of the same age (e.g., [7,43]). Second, as [41,42], it brings evidence of infants’ use of TP information when processing complex, natural language stimuli. Moreover, and to the best of our knowledge, this is the first study that establishes an effect on infants’ segmentation of manipulating the strength of the TP information present in the signal (since the comparison of Experiment 1 and 3 shows that manipulating TP strength affects the observation of syllabic segmentation).

Lastly, our results also show that both the rhythmic and the TP cues are used in combination by French-learning 8-month-olds, and that the relative strength of the two cues in the stimuli will determine the segmentation outcome (segmentation of a syllabic or bisyllabic word form). Similar effects had previously been found for stress-based English [15-18], and our results extend them for the first time to a syllable-based language, French. Note that a possible explanation, to be evaluated in future studies for the differences in performance found between Parisian and Canadian French infants might be related to differences in the relative strength of phonological (including rhythmic) cues and TP cues in these two dialects of French (see 10 for a discussion regarding the possible role of increased word-final accentuation in Canadian compared to Parisian French).

To conclude, the present study brings several important findings to our understanding of early segmentation processes. First, Experiment 3 establishes early syllabic segmentation (by 8 months) in syllable-based French, supporting the early rhythmic-based segmentation hypothesis, one of the two segmentation procedures that have been proposed to be crucial at the time of emergence of segmentation abilities [9]. Second, taken together, the present findings show that the use of the syllabic unit is done in conjunction with the use of transitional probability (TP) information, and that the segmentation outcome depends on the relative strength of both cues: when TPs are low, the segmentation outcome is the syllabic unit (Exp. 3); when TPs are highest, as in [10], the segmentation outcome is the bisyllabic word; when TPs are intermediate, no clear outcome is found, neither in terms of syllabic (Exp. 1) nor bisyllabic word (Exp. 2) segmentation. Future studies, using different kinds of methods (behavioral and/or ERPs), should keep exploring how infants use different cues to segment speech at different points in development and how the use of language-general statistical cues (TPs) and language-specific phonological cues (rhythmic units, phonotactic information, coarticulation, etc.) will lead to different trajectories of emergence of segmentation abilities according to the relative strength of these cues in different languages. In doing so, it will thus be important to expand the range of languages tested in the different rhythmic classes, taking care to set up experimental situations that allow researchers to dissociate the use of these various cues (e.g., TPs and rhythmic cues). This will in turn allow them to continue articulating the link, for word form segmentation, between the two influential theories for language acquisition explored here, namely the statistically-based and the prosodic/phonological bootstrapping theories.

## Supporting Information

Appendix S1 **Passages used in the familiarization phase of Experiments 1 & 2.**
(TIFF)

Appendix S2 **Passages used in the familiarization phase of Experiment 3.**
(TIFF)
