# Supplementary figures and images for: Early Syllabic Segmentation of Fluent Speech by Infants Acquiring French
Source: PLoS One. 2013 Nov 7;8(11):e79646. doi: 10.1371/journal.pone.0079646 (PMC3820683; doi:10.1371/journal.pone.0079646)

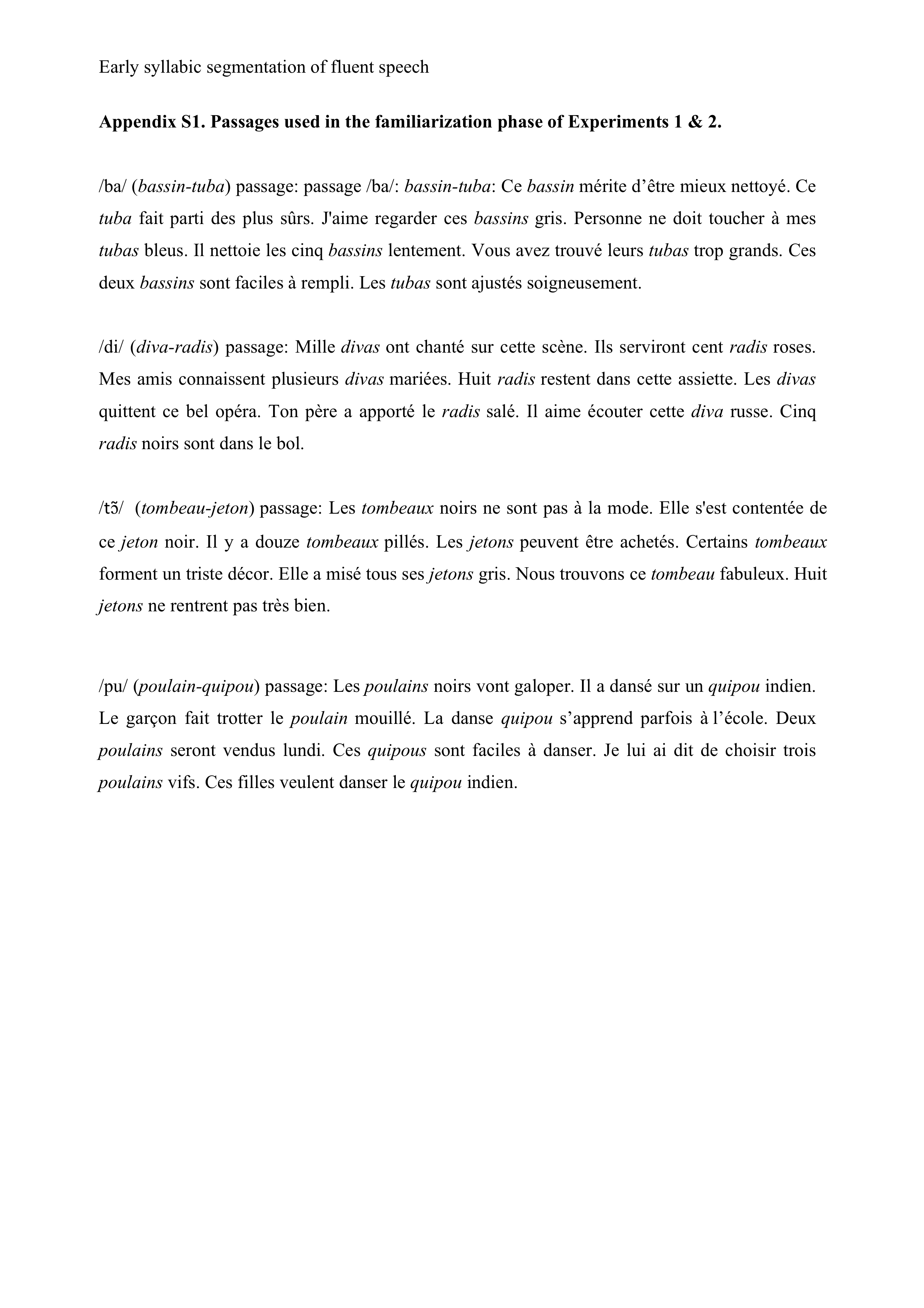

Supplement: Appendix S1 — Passages used in the familiarization phase of Experiments 1 & 2. (TIFF) [file pone.0079646.s001.tiff]

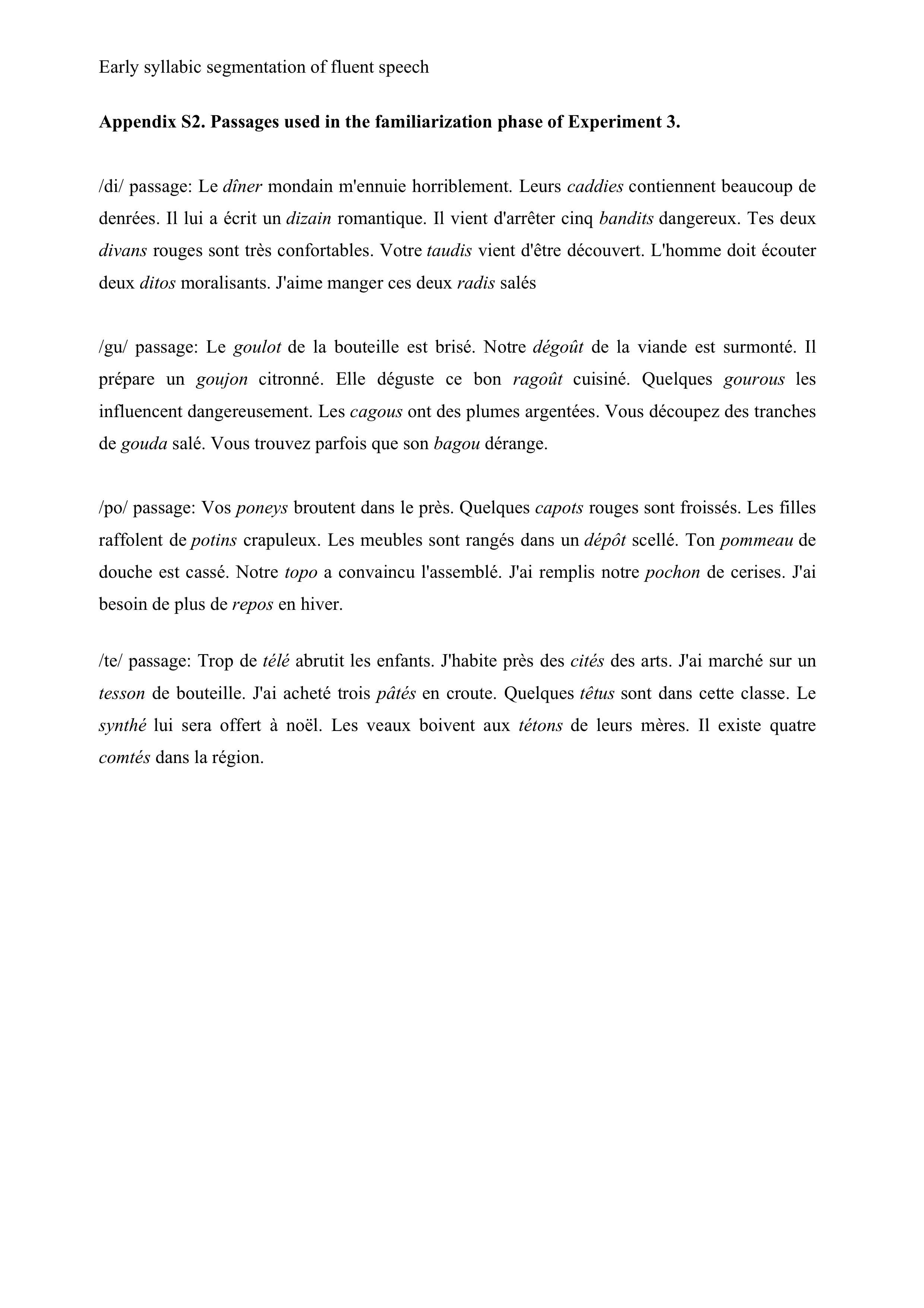

Supplement: Appendix S2 — Passages used in the familiarization phase of Experiment 3. (TIFF) [file pone.0079646.s002.tiff]
